# Editorial: Venous thromboembolism and pregnancy

**DOI:** 10.3389/fcvm.2022.1113941

**Published:** 2022-12-23

**Authors:** Bernard Tardy, Laurent Bertoletti

**Affiliations:** ^1^Clinical Investigation Center, Inserm CIC1408, Saint-Étienne, France; ^2^Université Jean Monnet, Department of Médecine Vasculaire et Thérapeutique, University Hospital of Saint-Etienne, Mines Saint-Étienne, INSERM, U 1059, CIC1408, Saint-Étienne, France

**Keywords:** pregnancy, post-partum, thrombosis, anticoagulant, pulmonary embolism

Around 700 women die each year in the U.S., from conditions related to or associated with pregnancy or childbirth (the highest rate among developed nations) (1), and over 50,000 women experience severe maternal morbidity (SMM) (2). In addition to this alarming finding, overall pregnancy-related mortality is increasing, and the scientific community is still unclear as to why this is occurring. The World Health Organization defines maternal morbidity as any health condition attributed to and/or aggravated by pregnancy and childbirth that has negative outcomes on the woman's wellbeing (3). As with maternal mortality (MM), maternal morbidity has also seen increasing numbers. Excluding blood transfusions, the rate of SMM increased by ~20% from 1993 to 2014 in the U.S. (2).

In response, the NIH Office of the Director (OD), the Eunice Kennedy Shriver National Institute of Child Health and Human Development (NICHD), ORWH, and other NIH Institutes, Centers, and Offices have developed the trans-NIH Implementing a Maternal health and PRegnancy Outcomes Vision for Everyone (IMPROVE) initiative to support research into how to reduce preventable MM; improve health for women before, during, and after delivery; and promote health equity in the United States. “Any maternal death is one too many,” said NICHD Director Diana W. Bianchi, M.D., co-lead of the IMPROVE Task Force. “Areas of research include heart disease, hemorrhage or bleeding, and infection (the leading causes of U.S. maternal deaths); contributing conditions, such as diabetes, obesity, mental health disorders, and substance use disorders; and structural and health care system factors that may contribute to delays or disruptions in maternal care.”

It is for this reason that we are pleased to see the appearance of this Research Topic, which provides a recent update on the various aspects of venous thromboembolism (VTE) risk in pregnant and postpartum women.

Pregnancy represents a unique situation, increasing the risk of thrombosis both throughout the pregnancy and during the postpartum period. The main risk factors have been clarified thanks to the epidemiological studies, summarized by Gris et al. It is thus possible to separate pre-existing risk factors, transient risk factors, and risk factors specifically associated with pregnancy. It is likely that with the change in populations and the development of medically assisted reproduction, the risk factors may change in the coming years. Thus, other studies such as the case-control study conducted by Alsheef et al. are welcome, in order to be able to inform possible changes in the epidemiology.

These epidemiological data have allowed the development of scores aimed at predicting the risk of venous thrombosis disease during pregnancy. Raia-Barjat, Chauleur et al. remind us of the main scores, as well as their different levels of validation. The individualization of patients with a higher risk of developing venous thromboembolic disease has made it possible to propose the implementation of a venous thromboembolic disease prevention strategy. Particular attention is paid by Blondon and Skeith to the prevention of venous thromboembolism during the postpartum period, which appears to be the period of greatest risk for thromboembolic events.

Despite prevention strategies, we still have to manage pregnant women with a suspicion of pulmonary embolism. Great progress has been made in recent years for these patients, who were previously excluded from the main diagnostic tests. Robert-Ebadi et al. examine the different algorithms currently validated, allowing physicians to reject the hypothesis of pulmonary embolism without the need for thoracic imaging.

Although the prevalence of pulmonary embolism is relatively low in pregnant women suspected of having a pulmonary embolism, specific situations such as high-risk PE may be challenging, as discussed by Hobohm et al., or for patients known to have antiphospholipid syndrome, as discussed by Killian and van Mens. The use of inferior vena cava filters is also a potential issue, as presented by Bistervels et al. It is important to note that the rate of complications directly related to the filter is nearly one woman in five.

The final article, by Raia-Barjat, Ni Ainle et al., deals with the problem of pre-eclampsia in the case of venous thromboembolic disease, recalling the vascular role of the placenta and the current discussions on the possibilities of its prevention.

As brilliantly illustrated by the HIGHLOW study (4), evaluating the efficacy and safety of an intermediate dose of low molecular weight heparin in the prevention of venous thromboembolic disease in high-risk women, being pregnant is no more a reason to not be included in trials. This opens the way to make progress on many aspects of VTE in pregnant and postpartum women. Thromboprophylaxis aims to protect patients from venous thromboembolism, but at the risk of an increased risk of bleeding, which may occur in different manners during pregnancy and post-partum (5). During the post-partum, the use of direct oral anticoagulants may also be challenging, as it is associated with an increased risk of genitourinary bleeding (6), a setting that may need dedicated assessment tools (7). In these situations, the potential of anti-Factor XI (8) deserves specific consideration. Notably, both DOACs and small peptides may not be used during lactation. Future work should also address the management of superficial venous thrombosis, as data are limited in the field (9). Finally, long-term follow-up is needed, to assess the risk of vascular sequelae, particularly pulmonary sequelae (10). We very sincerely hope you enjoy reading this Research Topic as much as we enjoyed accompanying the authors through production.

## Author contributions

Both authors listed have made a substantial, direct, and intellectual contribution to the work and approved it for publication.
